# Music Feature Extraction Method Based on Internet of Things Technology and Its Application

**DOI:** 10.1155/2022/8615152

**Published:** 2022-04-18

**Authors:** Bo Chen, Heung Kou, Bowen Hou, Yanbing Zhou

**Affiliations:** ^1^Lianyungang Normal College, Lianyungang, Jiangsu 222000, China; ^2^Department of Education, Graduate School of Sehan University, Yeongam-gun, Jeollanam-do 58447, Republic of Korea; ^3^Guangdong Medical University, Dongguan 523808, China

## Abstract

Due to the influence of factors such as strong music specialization, complex music theory knowledge, and various variations, it is difficult to identify music features. We have developed a music characteristic identification system using the Internet-based method. The physical sensing layer of our designed system deploys audio sensors on various coordinates to capture the raw audio signal and performs audio signal processing and analysis using the TMS320VC5402 digital signal processor; the Internet transport layer places audio sensors at various locations to capture the raw audio signal. The TMS320VC5402 digital signal processor is used for audio signal diagnosis and treatment. The network transport layer transmits the finished audio signal to the data base of song signal in the application layer of the system; the song characteristic analysis block in the application layer adopts dynamics. The music characteristic analysis block in the applied layer adopts dynamic time warping algorithm to acquire the maximal resemblance between the test template and the reference template to achieve music signal characteristic identification and identify music tunes and music modes based on the identification results. The application layer music feature analysis block adopts dynamic time regularization algorithm and mel-frequency cepstrum coefficient to achieve music signal feature recognition and identify music tunes and music patterns based on the recognition results. We have verified through experiments, and the results show that the system operates consistently, can obtain high-quality music samples, and can extract good music characteristics.

## 1. Introduction

With the rapid growth of computers and the Internet since the twenty-first century, audio is digitally recorded, edited, and produced. This has made it easy to access the desired number of songs stored in mobile music players and even thousands of tracks using online on-demand music media services. Such an enormous amount of music can no longer be managed by hand alone and requires new methods to describe, index, search, and interact with it, namely, content-based music information retrieval (MIR) [1–3]. This technology involves the intersection of several disciplines and has a wide range of application needs, such as aggregation. It is a worthwhile area of research in the fields of aggregation and dissemination of music, music retrieval, and music production.

Music identification is developed on the basis of the art of speech identification, where the music content is obtained from the audio signal and further musical characteristics such as musical style and emotion are obtained [4, 5]. The study of audio characteristic identification covers many areas, for example, instrument analysis, psychoacoustics, and music knowledge. Audio recognition systems are not widely used within modern music because of the absence of an overall system design framework adept at improving performance information. The implementation of audio characteristic recognition systems has been made possible by the advent of IoT [6]. IOT technology can realize the intelligent collection, processing, and analysis of music signals. It has the advantages of complete sensing, convenience, reliable transmission, and fast speed [7]. An audio characteristic identification system built on IoT is designed with the aim of sensing, transmitting, and recognizing music signals using IoT technology.

The general function of the Internet of Things is to collect light, sound, infrared, temperature, and other information in the environment through various sensors and upload these sensing information to the central control system, which will analyze and process these information and combine the research results of music psychology so as to select suitable music for the purpose of improving the mood of users and enhancing work efficiency [8]. Also, customers can contribute to the system by sharing their music data, commenting on songs, checking song messages, etc. on the website offered by the system [9]. With the quick growth of big data technology cloud computing [10, 11], AI [12, 13], and the Internet, technology has profoundly changed people's emotional perceptions, value orientations, ethical standards, thinking styles, and behavioral habits. Nowadays, it is extremely convenient for people to obtain information from the Internet, but it also brings about the trouble of screening useful information, and the phenomenon of “information overload” appears. A musical melody is a series of fundamental frequency levels which correspond to the pitches in the key. As the middle and high-level semantics of music, it can be used to describe music content and can be used as a frontend for related applications such as humming retrieval systems, music genre classification, and music emotion recognition. It makes the extraction of music melody and its application a research hotspot in the field of music information search.

As an important part of human life, music can convey emotions and regulate the emotions of listeners. Emotion is the essential feature of music, and the relationship between emotion and music has become the subject of many academic studies. The research spans many different fields, including philosophy, musicology, psychology, biology, anthropology, and sociology. The study of music melody extraction methods and their applications was first proposed by Goto in the 1990s and introduced by Emilia Gómez and Beesuan Ong to the ISMIR conference for discussion and as one of the MIREX reviews. Several review papers on music melody extraction methods have been published in China and abroad [14, 15], among which Salamon et al. [16] analyzed the 16 most representative melody extraction algorithms in MIREX reviews since 2005 and grouped melody extraction algorithms into three categories according to their characteristics: saliency-based, source separation-based, and data-driven [17]. Tone is the core element of music. The extraction and recognition of the basic features of sounds are the basic contents.

In the field of signal processing, a common frequency domain processing method is the Fourier transform. The Fourier transform projects the signal in the time domain to different individual frequency components in the frequency domain and thus analyzes the signal from the perspective of frequency. In the field of signal processing, the Fourier transform has a high status. However, the Fourier analysis method also has shortcomings. For example, Fourier analysis can only obtain the overall spectrum of a signal in the time domain at a macroscopic level, i.e., the total intensity of each frequency component of the signal. However, for these components, we cannot obtain information about the specific moment of their appearance. Therefore, the Fourier method cannot obtain the frequency characteristics of the signal near any moment, i.e., it cannot meet the requirements of analyzing time-varying nonstationary signals.

**Figure 1:**
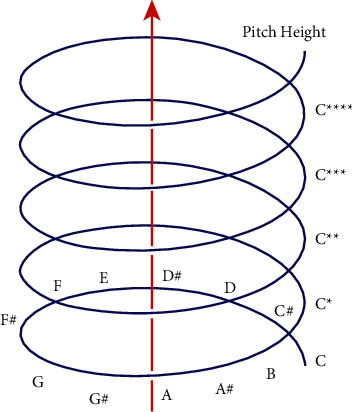
Spiral model of pitch.

Different notes can have different sensations when the pitch is doubled (i.e., pitch height [18]). However, music psychologists believe that there is another circular dimension of pitch dimension (i.e., pitch level or pitch chromaticity). In Eastern and Western music, the musical scale is based on this circular structure. If the scale is viewed in ascending order, it will go through a cycle of pitch levels, i.e., the scale goes from C, C#, all the way to B, then back to C, and so on. In summary, pitch is a 3D spiral with both vertical and circular dimensions, and in the successive rotations of the spiral, pitch is separated by octaves, as shown in Figure 1.

Pitch is a subjective perceptual quantity, and then there are differences in the perception of pitch by different people. For the same sound, different people may associate it with a sine wave with different frequencies, i.e., reflecting the variability of pitch perception. In order to represent the subjectively perceived pitch using an objective physical quantity, the pitch is related to the frequency. Frequency [19] is a measurable quantity that can be the reciprocal of the period for a periodic or near-periodic signal [20].

**Figure 2:**
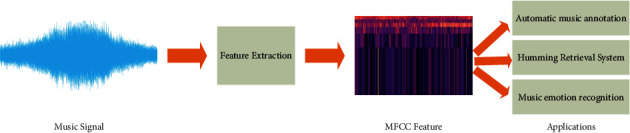
Music feature extraction and related applications.

Content-based MIR focuses on two aspects of audio content: the description of audio content, i.e., the automatic extraction of meaningful information from the audio signal, and the utilization of audio content, i.e., the use of the obtained audio content for various applications. The description and application of melody, one of the three elements of music, is an important research topic in the area of MIR. It aims to extract from the audio signal the sequence of fundamental frequency values relative to the pitch values of melodic lines of music, so that the extracted results can be applied to music information retrieval techniques related to melody, as shown in Figure 2.

Identifying the melody of music may be commonplace for an average listener. Even if one has not received any musical training since childhood, one can still easily hum the melody of our own favorite songs. Those who have received musical training can even write music to the melodies they hear. But when we try to automate the extraction of musical melodies in other ways, it becomes a very complex and challenging challenge. The reasons for this difficulty are twofold.

1. Polyphonic music signals are recorded by superimposing sound waves from all instruments, and these instruments are played simultaneously most of the time. Therefore, when studying the spectral content of the music signal, it is very tough to separate and correspond the spectra originating from different sound sources that are highly coupled together according to the target harmonic structure to individual notes. Also, the later reverberation and echo processing will further increase the degree of mixing of each sound source, blur the note endpoints in time, and make the extraction more complicated.
2. Even if the fundamental frequency representation of the music signal has been obtained, it is still necessary to determine which pitch values belong to the melody and which ones belong to the accompaniment. This judgment will be even more difficult when background harmonies are present in the music.

The music extraction method has many direct and indirect applications. Direct applications include the automatic labeling of music (music score generation), tonal analysis (analysis of interval relationships between pitches), melodic theme and pattern analysis (identification of musical styles), and other areas of computational musicology and music education. Indirect applications are mainly seen in its use as humming search (searching for target songs by humming), cover song identification, music genre classification (organizing songs according to genre), music emotion recognition, song elimination (removing vocals from music to obtain accompaniment for karaoke), music structure analysis (analyzing the composition), and singer identification as intermediate processing steps in the study. In addition, music melody extraction techniques are increasingly being integrated as plug-ins into music processing tools such as Adobe Audition, Sonic Visualiser, and Melodyne.

In the field of music information search, ISMIR is an annual international music academic conference. Since 2005, MIREX, which is held concurrently with the conference, has included audio melody extraction (AME) as one of its contest tests. In summary, music melody extraction has a broad application and is important for studies in the domain of music message search.

## 2. Overall Structure of Music Feature Recognition System

**Figure 3:**
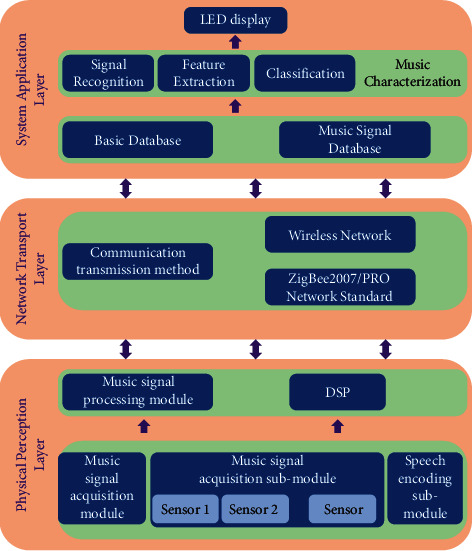
Block diagram of the overall system structure.

The system of music feature extraction method based on IoT technology mainly consists of physical sensing layer, network transmission layer, and system application layer together, and the overall system structure block diagram is shown in Figure 3.

The physical sensing layer mainly includes the music signal acquisition block and the music signal treatment block. The music signal acquisition block identifies the required music signal through the sound sensor acquisition system deployed at different locations and transmits the acquired music signal to the music signal processing block, which uses a DSP processor to process the music signal. The network transmission layer transmits the data collected and processed by the physical sensing layer to the system application layer by means of wireless network communication transmission. The system application layer gathers music signals to form a music signal database, and after music signal feature extraction and feature recognition classification in the music feature analysis block, the music feature recognition results are displayed through LEDs.

### 2.1. Design of Music Signal Acquisition Block

The music signal capture block includes a music capture subblock and a speech encoding subblock. The music acquisition subblock consists of sound sensors installed at different locations, which are responsible for collecting the original music signal. The sound sensor has a built-in sound-sensitive condenser electret microphone, which is used by the A/*D* converter and then the sound sensor has a built-in capacitive electret microphone sensitive to sound, which is converted by A/*D* converter and transmitted to the speech coding subblock. The speech coding subblock is mainly responsible for the original high-fidelity lossless compression of the original music signal, converting the music signal into transmittable data information and then transmitting it to the audio processing block.

### 2.2. Music Signal Processing Block Design

**Figure 4:**
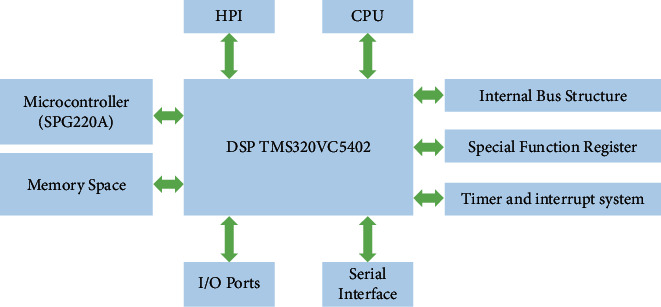
TMS320VC5402 functional structure diagram.

The audio signal processor module is engineered by a DSP processor. The block uses a fixed-point DSP chip of TMS320VC5402 DSP for speech signal processing, which has low power consumption and fast operation, carries two multichannel buffered serial ports (MCBSPS), can connect to compiler-decoder (CODEC) for speech input, has an 8 bit enhanced host parallel port (HPI8), and establishes a communication connection with the host computer. Its structure is shown in Figure 4.

The TMS320VC5402 internal function units are as follows. The internal bus structure consists of four address buses and four program/data buses to form eight 16 bit buses. The special function registers contain 26 special function registers for control, management, and access to each function unit. The timer and interrupt system itself carries a 16 bit timer with a 4 bit prescriptive scale. The TMS320VC5402 DSP memory has a basic space size of 192 kB, with program space, data space, and I/O space each accounting for 1/3 of the size, of which the program storage space can be expanded to 1 MB. The TMS320VC5402 DSP has two general-purpose I/O ports, BIO, and XF. In addition, the I/O space can be accessed to expand the I/O ports. The MCBSP of the TMS320VC5402 is capable of operating in SPI mode, which is beneficial for interfacing with serial A/*D* and serial E2PROM. The host port supplies a parallel interface for the DSP to connect with external processors, facilitating the exchange of information between the DSP and external processors.

## 3. Music Feature Recognition

### 3.1. Dynamic Time Warping

The music characteristic analysis block of the system application layer uses the dynamic time warping (DTW) algorithm for identifying music signal features by comparing the Euclidean separation between the music characteristic test and reference templates. The speech framing of the music characteristic test pattern and the reference pattern tracks follow the search route of the DTW algorithm to launch the music characteristic pairing. Assume that the resource pattern and test pattern are represented by

(1) $$\begin{array}{c} S=\left\{S\left(1\right),S\left(2\right),S\left(m\right),S\left(M\right)\right\},\\ P=\left\{P\left(1\right),P\left(2\right),P\left(n\right),P\left(N\right)\right\}, \end{array}$$

where the voice frames included in the reference template and the test provisional are denoted by *M* and *N*, respectively, and *m* and *n* are arbitrary frame numbers in *S* and *P*, respectively. The Euclidean range is computed by

(2) $$\begin{array}{c} l\left[P\left(n\right),S\left(m\right)\right]=\frac{1}{k}\sum_{\left(r=1\right)}^{K}{\left(H_{r}-H_{r}^{\prime }\right)}^{2}. \end{array}$$

Suppose that the paths pass through the lattice points in sequence as (*n*_*1*_*,m*_*1*_),...,(*n*_*i*_, *m*_*i*_), (*n*_*N*_, *m*_*M*_); according to the endpoint constraint, we can get (*n*_*1*_, *m*_*1*_)=(1,1), (*n*_*N*_, *m*_*M*_)=(*N*, *M*). To comply with the slope constraint, we set the slope selection interval to 0.5 to 2.5.

From the local search perspective, assuming that the previous grid point of the best path passing through (*n*_*i*_, *m*_*i*_) is one of (*n*_*i*_−1, *m*_*i*_) and (*n*_*i*_−1, *m*_*i*_−2) and assuming that the partial cumulative distances of the origin point from these three grid points are *L*[(*n*_*i*_−1, *m*_*i*_)], *L*[(*n*_*i*_−1, *m*_*i*_−1)], and *L*[(*n*_*i*_−1, *m*_*i*_−2)], then (*n*_*i*_, *m*_*i*_) picks the part of the minimum accumulated distance grid point to continue, and so on. The final path cumulative distance can be expressed by

(3) $$\begin{array}{c} L\left[\left(n_{i},m_{i}\right)\right]=l\left[T\left(n_{i},R\left(m_{i}\right)\right)\right]+L\left[\left(n_{i}-1,m_{i}-1\right)\right]. \end{array}$$

Thus, the minimum accumulated separation is the maximal value of the resemblance between the test template and the reference pattern, which is the result of the audio signal characteristic identification.

### 3.2. Mel-Frequency Cepstrum Coefficient (MFCC)

MFCC is a speech signal characteristic proposed by Mermelstein et al. in 1980 according to the features of human hearing, which exhibits a nonlinear correlation with frequency. With the intensive research on speech signals, mel inverse spectral coefficients have proved to be one of the most successful feature descriptions in speech recognition and detection applications internationally. All speech classes are basically studied on the basis of mel-frequency cepstrum coefficients. In sound processing, mel inverse spectral coefficient is a nonlinear mel scale frequency based on a linear cosine transform of the log power spectrum, and all speech classes are basically studied on the basis of mel-frequency inverse spectral coefficients. In sound processing, mel inverse spectral coefficient is a nonlinear mel scale frequency of log power spectrum based on linear cosine transform, reflecting the acoustic characteristics of the power spectrum. Mel inverse spectral coefficients take into account the characteristics of human ear recognition and simulate the process of sound propagation in the ear organ, which triggers blocking effect while ensuring the attenuation of external sound. In this paper, mel inverse spectral coefficients are chosen considering the principle of masking effect, i.e., bass tends to mask treble and treble has difficulty in masking bass, and the traveling distance of low-frequency sound waves on the inner ear basilar membrane is greater than that of high-frequency sound waves. Moreover, mel cepstrum coefficient can well enhance the high-frequency sound and prevent it from being masked by the low-frequency sound, and this property is consistent with the characteristics of human ear recognition.

MFCC which is a spectral parameter drawn in the frequency domain of the mel scale depicts the nonlinear characteristics of the frequency of the human ear, and its frequency dependence can be approximated by the following equation:

(4) $$\begin{array}{c} \text{mel}\left(f\right)=2595\times \mathrm{lg}\left(1+\frac{f}{700}\right). \end{array}$$

**Figure 5:**
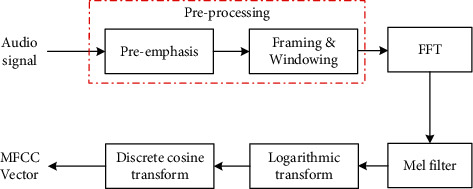
The MFCC extraction processing.

The extraction process of MFCC is as follows.  Figure 5 shows the MFCC extraction processing.

#### 3.2.1. Pre-Emphasis

Pre-emphasis processing is in fact the passing of the speech message via a high-pass filter.

(5) $$\begin{array}{c} \text{H}\left(Z\right)=1-\mu z^{-1}. \end{array}$$

Usually, the value of *μ* is taken between 0.9 and 1.0, and we usually take it to be 0.97. The aim of pre-emphasis is to raise the high-frequency portion so that the spectrum of the signal is kept flat over the full frequency range from low to high frequencies, and the spectrum can be sought with the same signal-to-noise ratio. This is also to remove the effect of the vocal folds and words when they appear, to make up for the high-frequency part of the speech message that is inhibited by the articulatory system, and also to accentuate the higher frequency resonance peaks.

The MFCC extraction process of music audio features does not require framing and windowing operations. The reason is that speech recognition systems need short-time features of speech signals to match speech input to text or words. The music feature MFCC extraction system has different requirements. The music feature MFCC extraction process looks more at the frequency composition in the music spectrum over a period of time rather than the short-time frequency characteristics. Therefore, the music feature MFCC extraction system does not need to precisely match two music signals on a time scale. Rather, it needs to describe the frequency composition of a music signal.

#### 3.2.2. Splitting Frames

First, N sample points are collected in an observation cell called a frame. Usually, the value of N is 256 or 512 and the coverage time is about 20 to 30 ms. In order to avoid variation between two adjacent frames, an overlap region is therefore made between the two adjacent frames, and this overlap region contains *M* sampling points; usually, the value of *M* is about 1/2 or 1/3 of *N*.

#### 3.2.3. Adding Window (Hamming Window)

In order to increase the signal frame continuity between the left and right sides, each frame of the signal is multiplied by the Hamming window. Suppose that the signal after framing is *S*(*n*), *n* = 0,1, ..., N-1, where N is the frame size; then, after multiplying the Hamming window, the form of *W*(*n*) is as follows:

(6) $$\begin{array}{c} W\left(n,a\right)=\left(1-a\right)-a\times \;\cos\left[\frac{2\pi n}{N-1}\right],\;0\leq n\leq N-1. \end{array}$$

### 3.3. Fast Fourier Transform

Because the characteristics of the signal are difficult to observe in the time domain, we often have to transform the signal into an energy distribution in the frequency range so that different energy distributions can be easily observed, which can represent the characteristics of different speech sounds. Therefore, each frame of the signal must be fast Fourier transformed and then multiplied by the Hamming window to obtain the energy distribution on the frequency spectrum. The spectrum of each frame is obtained from the FFT of each frame after opening the window. The power spectrum of the speech signal can be obtained by squaring the mode of the speech signal spectrum. The DFT of the voice signal varies as follows.

(7) $$\begin{array}{c} X_{a}\left(k\right)=\sum_{n=0}^{N-1}x\left(n\right)e^{-j2\pi k/N},\;0\leq k\leq N, \end{array}$$

where *x*(*n*) denotes the input data and *N* denotes the points of the FFT.

## 4. Experimental Analysis

**Figure 6:**
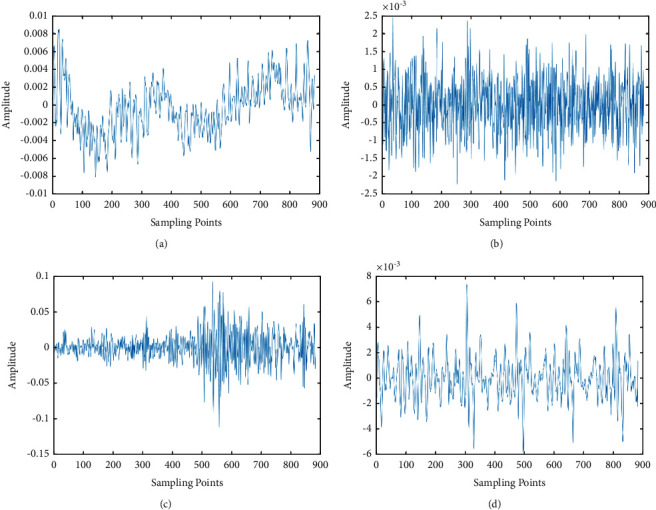
The original signals. (a)–(d) denote different kinds of raw signals, respectively.

**Figure 7:**
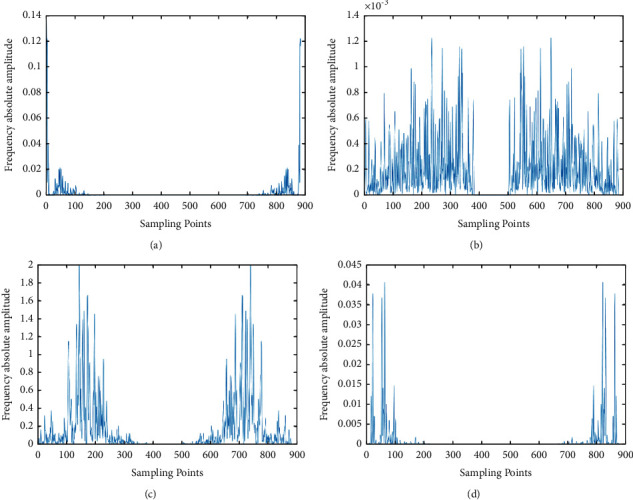
Frequency magnitude magnitudes. (a)–(d) denote the frequency replication magnitudes of each of the four different species.

**Figure 8:**
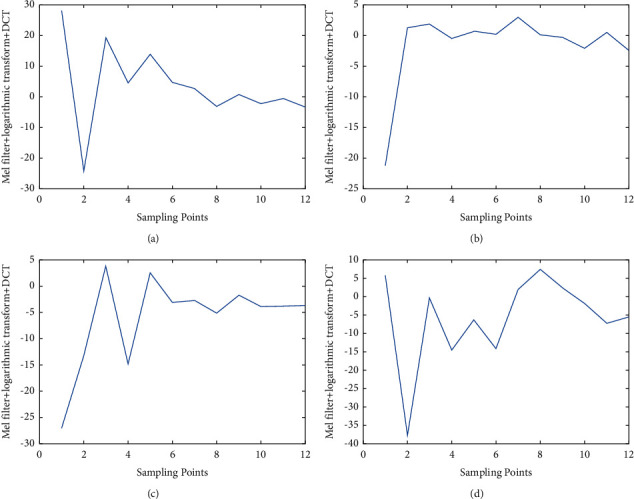
The result of the signal filtered by mel filter, logarithmic transform, and DCT variation. (a)–(d) denote the results for each of the four different signals.

**Figure 9:**
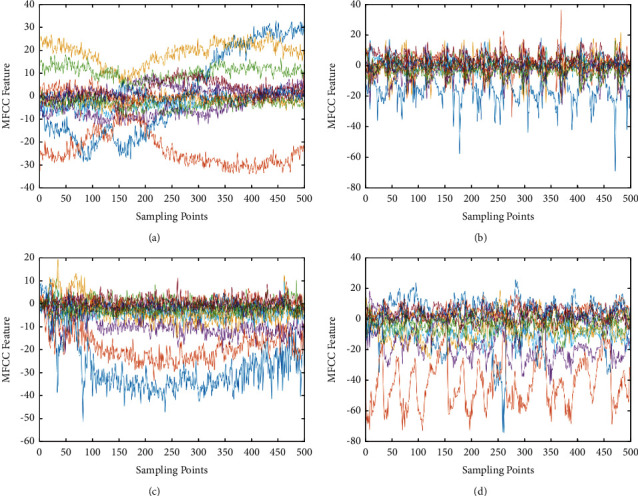
The MFCC characteristics of the signals. (a)–(d) denote the MFCC characteristics of the four different signals, respectively.

First we extract four different audio signals, and the original signals are shown in Figure 6. We can see that the original values of the different audio signals are different. According to the steps of MFCC feature extraction, we show the results of different processes in the follow-up. We put the four signals through Fourier transformation, and the results are shown in Figure 7. After the signal is filtered by mel filter, logarithmic transform, and DCT variation, the result is shown in Figure 8.  Figure 9 represents the MFCC characteristics of the four different signals. From the figure, we can see that the MFCC of different audio signals is very different, and the types of signals can also be judged by the naked eye.

Visual C++ was used to simulate the system prototype on Windows 10 platform to verify the effectiveness of the method proposed in this paper. The results of using this system to identify music features are shown in Table 1.

From the table, we can see that the system we designed can achieve feature recognition accurately, and the accuracy of recognition is largely determined because of the accuracy of feature extraction.

## 5. Conclusion

We have developed a music feature recognition system using an Internet-based approach. The audio signal is processed and analyzed by the TMS320VC5402 DSP. We perform feature extraction on the audio signal to extract its MFCC features. The features are used as input to the system, which eventually enables the recognition of music tunes and patterns. In our work, the accuracy rate of music feature recognition can reach 100%, which shows that our system can accurately recognize music features.

## Figures and Tables

**Table 1:** Music feature recognition results.

Types of music	Accuracy (%)
*a*	100
*b*	100
*c*	100
*d*	100

## Data Availability

The data used to support the findings of this study are available from the corresponding author upon request.
